# Qualitative comparative analysis of policies implemented by 26 European countries during the 2020 great lockdown

**DOI:** 10.3389/fpubh.2024.1396013

**Published:** 2024-08-12

**Authors:** Zhipeng Wang, Guangyi Qu

**Affiliations:** ^1^School of Marxism, East China University of Political Science and Law, Shanghai, China; ^2^Institute for Foreign-related Rule of Law, East China University of Political Science and Law, Shanghai, China

**Keywords:** COVID-19 pandemic, the 2020 great lockdown, party system, state fragility, decision-making

## Abstract

**Introduction:**

During the first wave of the COVID-19 pandemic in Europe, from March 1 to April 15, 2020, significant variations emerged among countries regarding the implementation of lockdown policies. During this period, viewed strictly from an epidemiological perspective, lockdown measures are considered the most effective means of containing a pandemic. However, the adoption of such measures varied, raising questions about whether the reluctance or failure of countries to implement lockdown policies reflected a disregard for epidemiological knowledge or stemmed from an inability to enforce these measures.

**Methods:**

This article employs Qualitative Comparative Analysis (QCA) with 26 European countries as case studies to investigate under what combination of conditions a country would implement lockdown policies.

**Results:**

The QCA results identify three distinct combinations of conditions that lead countries to implement lockdown measures. First, countries with relatively concentrated political power are more likely to implement lockdown policies. Among the 10 countries governed by a majority party or majority coalition within a two-party or moderate multi-party system, seven implemented lockdown policies. Second, in cases of relatively dispersed political power, countries facing state fragility risks are more likely to implement lockdown policies. Among the eight countries that meet both conditions, five implemented lockdown policies. Finally, factors such as political heritage, severity of the pandemic, demographic composition, healthcare access, quality standards, and the ruling party’s ideology play a lesser role in the decision to enact lockdown measures.

**Discussion:**

This article offers a novel perspective on the dynamics of party politics and state capacity in the context of decision-making during the COVID-19 pandemic. It contributes to a deeper understanding of the intricate relationship between political systems and public health crisis management, highlighting how various political and governance factors influence the adoption of public health interventions during crises.

## 1 Introduction

The concept of a “risk society” (1) has emerged as a key feature of today’s modern society, with frequent public crises serving as a primary manifestation of this phenomenon. Consequently, the governance of public crises has become a central theme in contemporary social science research. Uncertainty about risk stemming from insufficient knowledge present a more significant challenge to public governance than risks associated with predictability, which are defined by probabilistic processes (2). Notably, the COVID-19 pandemic, as a public health emergency, is typical uncertainty about risk. Thus, the research of crisis management strategies for the COVID-19 pandemic presented in this article holds significant real-world relevance. The outbreak of the COVID-19 pandemic at the close of 2019 has resulted in 772,386,069 reported cases to the World Health Organization (WHO), with 6,987,222 deaths up until December 13, 2023 (3). It is classified as a Public Health Emergency of International Concern (PHEIC) (4), characterized by a global pandemic (5) and the worst global crisis since World War II (6). During the first wave of the pandemic, owing to the lack of effective therapeutics in the management of the pandemic, non-pharmacological interventions such as travel bans, school and workplace closures, isolation of infected persons, quarantine of contacts, social distancing, and canceling mass gatherings were established as effective methods to control the spread (7, 8). Nonetheless, these measures presented significant practical and ethical challenges, forcing governments to balance safeguarding public health and economic-social development (9) while respecting the right to privacy and ensuring public interests, requiring tough decision-making and compromises (10). On one hand, the COVID-19 pandemic, a PHEIC, has provided a valuable case for examining public health crisis decision-making due to the complex environment confronted by policymakers in the first wave of the pandemic. On the other hand, the policy measures and their intensities adopted by different countries in response to the COVID-19 pandemic varied significantly, particularly during the first wave. This variation provides a basis for researching the decision-making process and influencing factors in public health crises. Therefore, this article focuses more on the reasons behind these policy differences rather than evaluating the effects of each country’s policies.

The Great Lockdown refers to the period of global economic slowdown and the implementation of widespread lockdown measures in response to the COVID-19 pandemic. Starting in early 2020, governments around the world imposed strict restrictions on movement, social gatherings, and economic activities in an effort to curb the spread of the virus. The International Monetary Fund (IMF) described the global economic downturn caused by the pandemic as the worst since the Great Depression of the 1930s. However, the term “The Great Lockdown” is specifically used to emphasize the role of lockdown measures in this economic crisis, distinguishing it from other economic downturns that were primarily driven by financial market failures or policy missteps (11). Europe stands as one of the most advanced areas globally in terms of politics, economics, and society. It is recognized as the cradle of modern states and boasts a high governance quality. Out of 38 OECD Member countries, Europe accounts for 26 (12). In relation to the COVID-19 pandemic, Europe reported 277,379,680 confirmed cases and 2,257,825 deaths to the World Health Organization (WHO) as of December 13, 2023. The confirmed cases in Europe make up 35.9% of the global count, while the death toll represents 32.3% of the global figure. After enduring the pandemic for over 3 years and with advancements in therapeutics, the situation is no longer considered a PHEIC. As a result, countries are transitioning to long-term management of the pandemic (13). However, during the initial wave of the COVID-19 pandemic in 2020, there were significant variations in COVID-19 policies among European countries. One specific area of variation was the policy of internal mobility restriction, which highlighted the decision-making dilemma. Even within the European Union (EU), different countries implemented varying levels of restrictions. This article aims to explore the factors contributing to these policy divergences. The focus of this article is on 25 EU countries and the United Kingdom (UK), excluding Malta and Luxembourg, totaling 26 European countries.

The findings of this article reveal that during the first wave of the pandemic, decision-making regarding mobility restriction policies in European countries shifted predominantly toward political considerations rather than scientific ones. The implementation of lockdown measures became the preferred strategy for those countries with centralized party systems or the risk of state fragility. During this decision-making stage, the severity of the pandemic and the ensuing public health implications for the country were not the foremost considerations. In essence, the question was not whether a country should implement lockdown measures but whether it was capable of doing so. This article provides a novel perspective on party politics and state capacity in the context of decision-making during the COVID-19 pandemic, contributing to a more nuanced understanding of the interplay between political systems and public health crisis management.

This article is structured into five main sections. First, it introduces the background, issues, and key findings. Second, the article provides a literature review and formulates an analytical framework. Third, it describes the methodology, case studies, and data used in this article. Fourth, it utilizes Qualitative Comparative Analysis to analyze the factors and principal pathways influencing the differences in mobility restriction policies among European countries during the first wave. Finally, it concludes by highlighting the implications of the findings.

## 2 Literature review and analytical framework

### 2.1 Literature review

During the first wave of the COVID-19 pandemic, countries globally were confronted with a PHEIC. At this stage, the timing and manner of implementing lockdown measures became the most critical public policy issues for governments in responding to the pandemic. According to scholars like Michael Howlett, the policy process can generally be divided into five stages: Agenda-setting, Policy formation, Decision-making, Policy implementation, and Policy evaluation (14). During this stage, the urgency of the pandemic made the stages of agenda-setting and policy formation uncontroversial, while the suddenness of the outbreak rendered policy implementation and evaluation impractical, thereby making the decision-making stage the focal point for studying mobility restriction policies.

Existing research indicates the decision-making tendencies of countries at this stage were influenced by multiple factors such as science, economy, socio-culture, international relations, and politics. From a scientific perspective, the lack of effective therapeutics for combating COVID-19 and the public’s trust in scientists over politicians (15) led to a significant influence of evidence-based decision-making led by public health experts and institutions (16). Scientific decision-making tools such as statistics (17), sociology, behavioral science (18), mathematical models (19), infectious disease models (20), health assessments (21), implementation science (22), citizen science (23), ambiguity reduction, and decision analysis (24), as well as big data and AI (25), became essential decision-making tools for governments. Secondly, the lockdown measures accrued economic costs. Due to the absence of comprehensive information about their healthcare system’s response capabilities, economic costs significantly influenced governments’ decision-making (26). Thirdly, from a socio-cultural perspective, the public’s acceptance of lockdown measures became a consequential factor influencing government decisions (27). The extent of acceptance is determined by the underlying socio-cultural factors. For instance, research shows that compared to American citizens who prioritize individual freedom and ignore official advice, while the Japanese people tend to adhere to government recommendations. Socio-cultural differences influenced decision-making and resulted in more effective policies in Japan (28). Fourth, the COVID-19 pandemic represents a PHEIC, necessitating cognitive coordination and collective action across countries (29). Consequently, international and regional organizations also influenced the decision-making of countries. The WHO, by informing members of existing scientific findings and persuading them to form a collective understanding, helped them surpass their inherent preferences to implement decisions conducive to controlling the pandemic (30). In Europe, the EU also exerted substantial influence. While public health policy is generally within the purview of Member states, in response to COVID-19, the European Council and the European Commission early on defined it as a public health crisis, thereby broadening the EU’s agenda to address the pandemic (31). The aforementioned literature has deepened our understanding of the decision-making environment during the first wave of the COVID-19 pandemic. However, these literature has not thoroughly explored the actual decision-making process. It is possible to identify the outcomes that the individual factor tend to produce, but the diverse and complex actual decision-making environment may lead to different decision outcomes of these factors under various combinations of conditions. Therefore, it is essential to conduct comparative studies of multiple cases to analyze decision outcomes under these various condition combinations.

Indeed, political factors are crucial in shaping governmental decisions in the pandemic decision-making process. This topic is mainly discussed across four dimensions. The first level pertains to the influence of individual politicians. In the first wave, the complex and pressing decision-making environment required politicians to make swift decisions. During such urgent decision-making, the predominance of emotions over rationality can easily result in excessive responses in the decisions made (32). The second dimension is the influence of political structures. Tensions in the power structure between central and local authorities in pandemic response also transmit to government decision-making. For instance, the federal systems in the United States (33) and Canada (34) make it challenging for central governments to implement efficient and unified pandemic containment decisions. The structure of power within the central government affects the efficiency of its decision-making. In Belgium, the cabinet, formed by a temporary minority during a significant crisis, was unable to enact effective and prompt lockdown measures (35). Party politics, especially partisan bias, impacts containment decisions at multiple levels, a phenomenon that has garnered considerable academic focus in the United States (36). The aforementioned research has also prompted this article to pay attention to the power structures of governments and party politics in various countries. The third dimension relates to the impact of political systems. In the discussion of the impact of political regimes on pandemic decision-making, the pros and cons of democratic and authoritarian systems are revisited, yet there remains no definitive conclusion (37–41). The fourth dimension addresses the role of constitutional courts in judging the constitutionality of pandemic response decisions. All national constitutions incorporate provisions that enable governments to implement emergency measures to address public health emergencies effectively. Nonetheless, the application of different judicial review approaches and criteria by constitutional courts worldwide leads to varied constitutionality rulings on identical pandemic containment measures (42). For instance, while the constitutional courts of Germany (43) and Belgium (35, 44) have deemed stringent lockdown measures constitutional, Spain’s Constitutional Court has ruled them unconstitutional (45).

Political factors have a more direct relationship with the public crisis decision-making process. The existing literature mainly analyzes the impact of political factors from two dimensions: individual and structural. However, both dimensions have their limitations. Individual factors, while highly dynamic, are challenging to form effective explanations that align with the principles of empirical social science. Conversely, structural factors interpret decision outcomes as reflections of political structures, overlooking the dynamic elements within the decision-making process. The previous research, particularly in comparative studies of COVID-19 policies, has noted the influence of party politics on crisis decision-making but has been limited to case studies without in-depth multi-case comparative analysis (46). Therefore, to address the aforementioned issues, it is significant and effective to conduct a further multi-case comparative analyses on the role of political parties.

### 2.2 Analytical framework and the conditions

**Figure 1:**
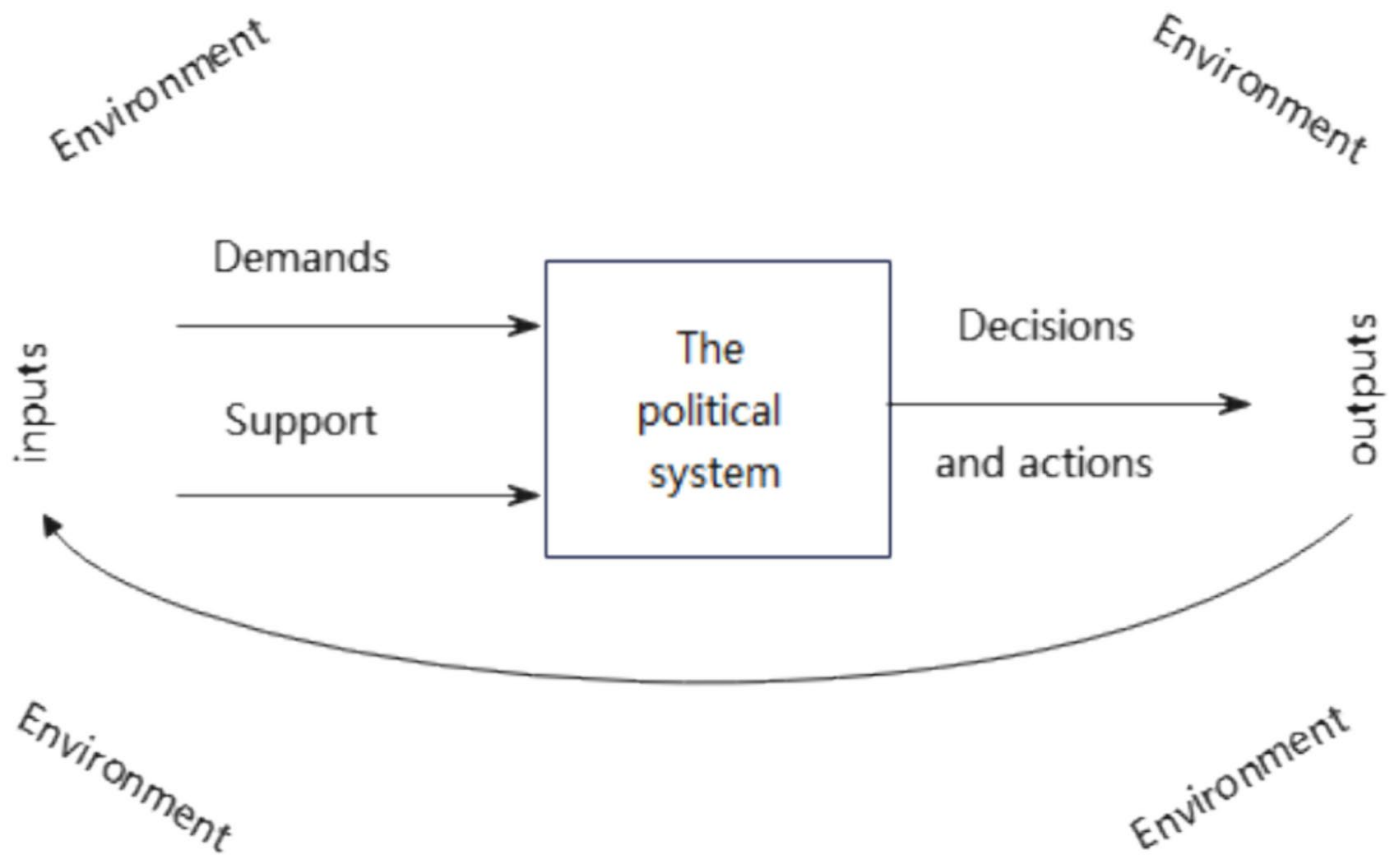
A simplified model of the political system decision-making. Source: Easton (47).

The political system decision-making model is a significant theoretical framework in public policy research. This model emphasizes the relationships, interactions, and results between the policy environment, the political system, and decisions. David Easton, in his research on political systems, developed a more refined theoretical model for decision-making in political systems. The simplified system of this theoretical model is illustrated in Figure 1.

Originating from this theoretical model, the development of public policy can be understood through three interconnected phases. The first phase is the inputs of demands and supports from the policy environment, which consists of internal societal factors such as ecology, biology, individuals, and social systems, as well as external aspects like international politics, international ecology, and international social systems, creating “the flow of effects” on public policy and resulting in particular demands and support for public policy. The second phase is the response of the political system to policy inputs, involving the process of strategic negotiation and weighing by policy agents within a given political system upon receiving these inputs. The third phase involves the outputs of public policies, namely the creation of specific public policies following the reaction of the political system, which then exposes an impact on the surrounding policy environment. These three phases constitute the entire process of public policy formation and create a feedback loop between inputs from the policy environment and outputs of public policy (47).

As a vital public policy for responding to public health crises, mobility restriction policies can be analyzed by using the aforementioned model to understand their policy processes and mechanisms. Based on this theoretical model, as this article focuses on the differences in public decision-making without analyzing the policy effects it produces, we can understand the mobility restriction policies through two main links. The first link involves inputs of the policy environment. This specifically refers to the impact of a series of political, economic, and social factors that constitute the policy environment in different countries on the necessity to implement mobility restriction policies. The second link is the response of the political system. More specifically, it is the impact of the political systems of various countries on the decision-making.

Hinged on the aforementioned theoretical model and existing research, the factors that may influence the implementation of mobility restriction policies can be primarily categorized into two types. The first category comprises seven factors that constitute the pandemic containment policy environment: “political heritage (whether a former Eastern Bloc country),” “severity of the pandemic,” “healthcare access and quality,” “demographic composition,” “economic situations,” “quality of the democratic regime,” and “state fragility.” The second category includes five factors that make up the political system for decision-making in mobility restriction policies: “political system,” “forms of government,” “party system,” “forms of cabinet,” and “the ruling party’s ideology.”

**Figure 2:**
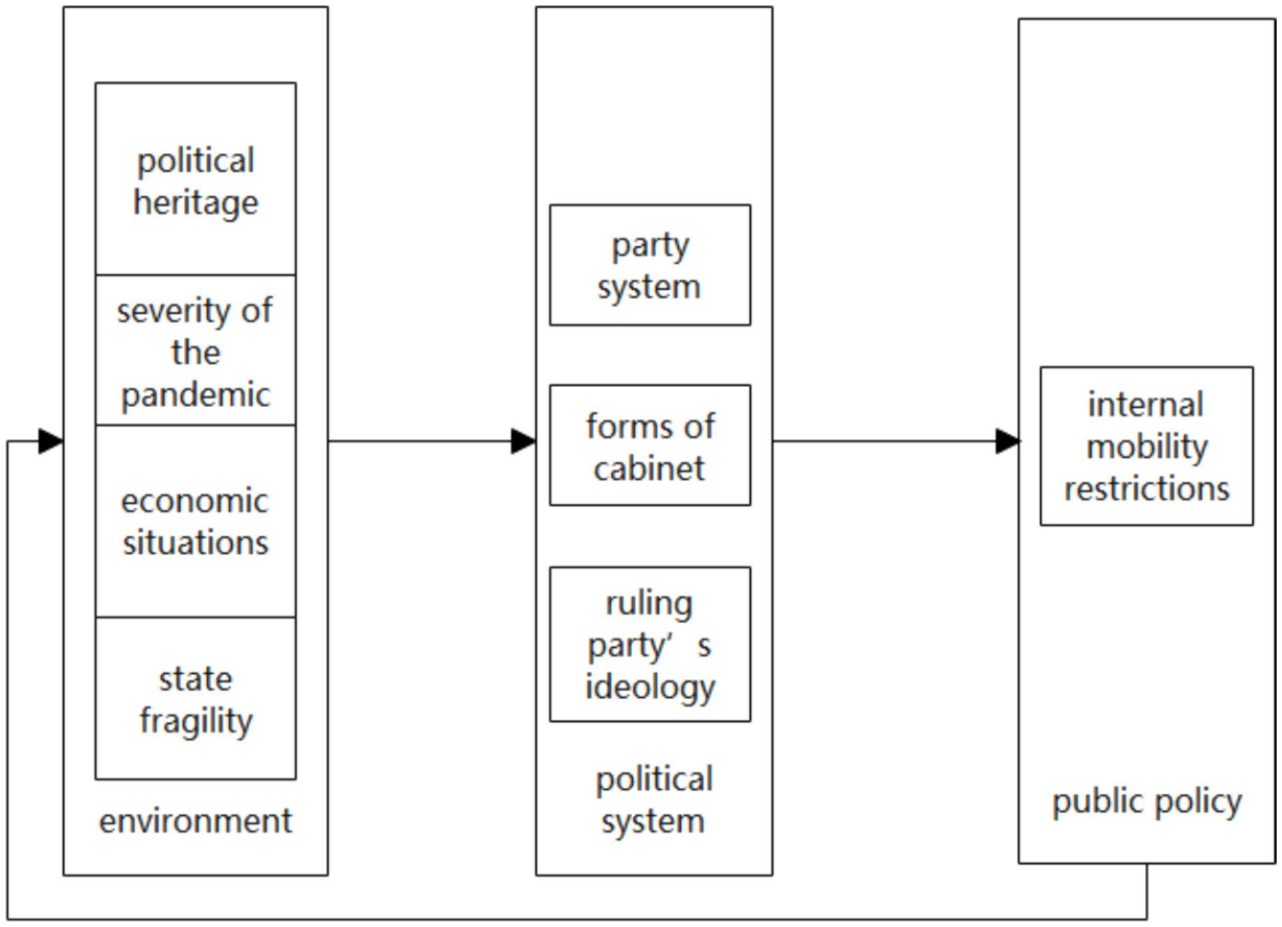
Analytical framework.

Based on the circumstances of the cases regarding the aforementioned influencing factors, there are no significant differences in “Healthcare Access and Quality Index” (48), “demographic composition” (49), “quality of the democratic regime” (50), “political system,” and “forms of the government” among the cases. Overall, they exhibit highly similar characteristics. Upon preliminary comparative analysis of these factors with mobility restriction policies, there is no relevance between them. Therefore, these factors are not included in the subsequent analysis of this article. Predicated on the existing theories, this article proposes the following Analytical Framework, focusing on seven factors that may affect the mobility restriction policies (Figure 2).

## 3 Research design and methodology

### 3.1 Cases and data sources

The Robert Schuman Center for Advanced Studies released a database (CMMP-A) on the basic situation of population mobility restriction policies implemented by 32 European countries in response to the COVID-19 pandemic from March 1 to May 31, 2020 (51). The database collects both internal mobility restrictions and external / cross-border mobility restrictions. Since the policy intensity of implementing internal mobility restrictions is superior to external / cross-border mobility restrictions, and the impact on the economy and society is generally greater, as a result, the internal mobility restrictions in the database can be used to measure the strength and basic situation of the mobility restriction policies of various countries. As shown in Table 1, the database distributes the internal mobility restrictions into four levels according to their policy intensity.

**Table 1:** CMMP-A database for the period from March 1 to May 31, 2020 code rules for internal mobility restrictions in Europe.

Values of limitations	Type of restriction	Limitations
0	Unrestricted mobility	No restrictions
1	Limited mobility	Population recommended to limit unnecessary movement, some limitations on businesses, some educational institutions closed, some limitations for public or private events, mild restrictions on public gatherings
2	Minimal mobility	Population asked to stay at home, most businesses closed, most educational institutions closed, cultural and private events severely restricted, public gatherings severely restricted, transport within the country limited, travel between regions limited
3	Lockdown	Mandatory stay-at-home orders with curfews and very limited exceptions in addition to measures listed under level 2

Regarding case selection, first of all, in order to further control the differences in policy environment among the cases, this article will focus on the EU countries in the database. Second, considering that the small population size may amplify the impact of certain specific factors on public policy, we will exclude Malta and Luxembourg whose population are less than 1 million. Finally, the UK withdrew from the EU (Brexit) on January 31, 2020, which was close to the first wave of the Covid-19 pandemic. Regardless of this fact the UK is still included in this article due to its significance as an important European country. In brief, the cases of this article mainly focuses on 25 EU countries and the UK except Malta and Luxembourg, resulting in a total of 26 European countries.

**Figure 3:**
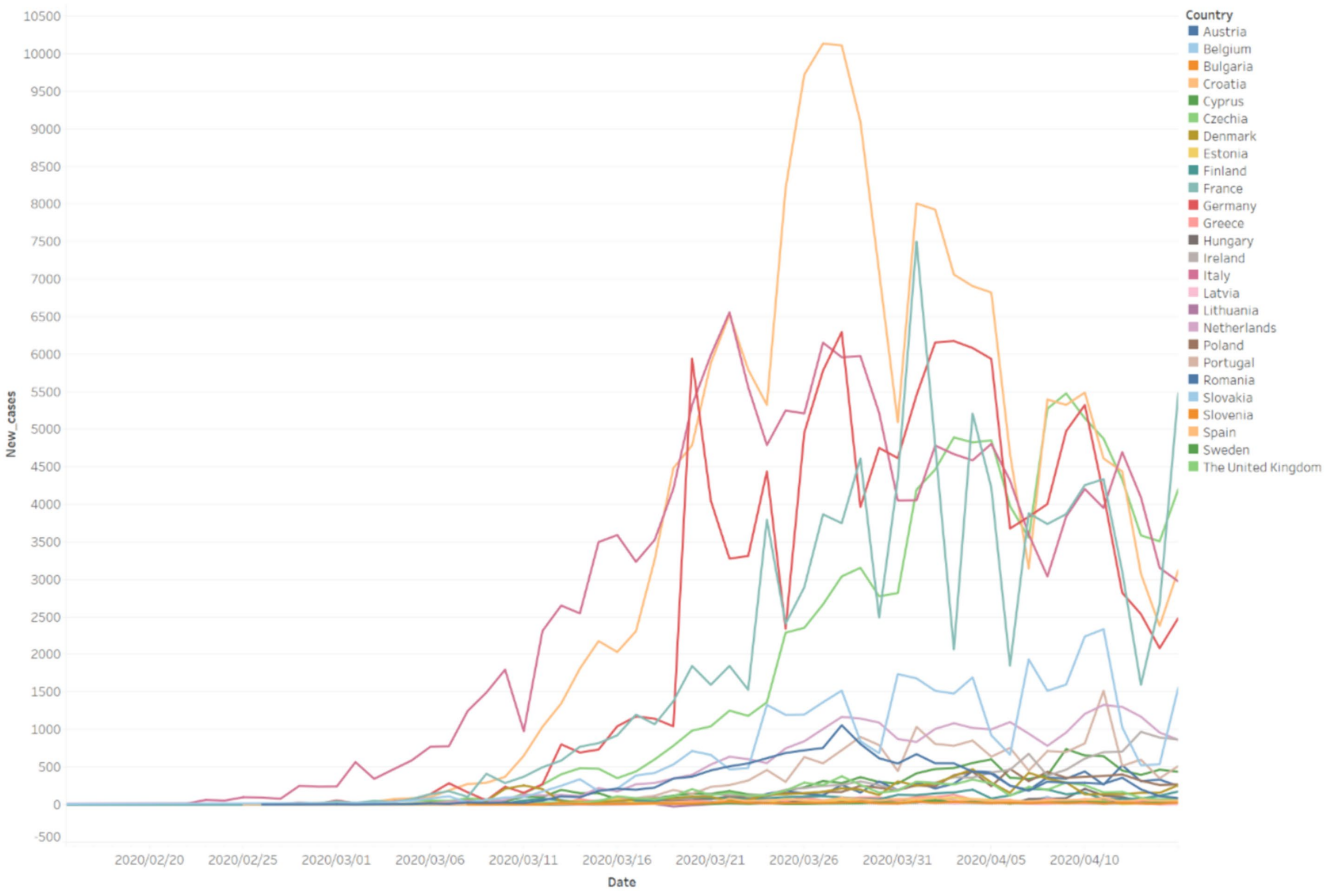
New COVID-19 cases were confirmed in 26 European countries before April 15, 2020.

In terms of the time range, on the one hand, most of the first confirmed cases of COVID-19 in the countries occurred from the end of February to the beginning of March, so March 1 can be deemed to be the point for the outbreak of COVID-19 in European countries. On the other hand, as shown in Figure 3, in this round of pandemic transmission in the countries, the turning point of the pandemic in most countries occurred from the end of March to the middle of April. Therefore, April 15 can be regarded as the point when the first wave in European countries comes to an end. To sum up, the time range of the first wave in European countries is from March 1 to April 15.

Through the analysis of the implementation of internal mobility restrictions in 26 European countries during this stage, it is identified that, as shown in Table 2, we will code “1” for countries that have implemented lockdown measures, that is, countries with level 3, and code “0” for countries that have not implemented them during the period from March 1 to April 15, 2020. We discovered that out of 26 countries, 14 have implemented lockdown measures and 12 countries have only implemented internal mobility restrictions below level 2.

**Table 2:** Implementation of “lockdown” in 26 European countries from March 1 to April 15, 2020.

Country	Lockdown
AUT	1
BEL	1
BGR	0
HRV	0
CYP	1
CZE	1
DNK	0
EST	0
FIN	0
FRA	1
DEU	0
GRC	1
HUN	0
IRL	1
ITA	1
LVA	0
LTU	0
NLD	0
POL	1
PRT	0
ROU	1
SVK	1
SVN	1
ESP	1
SWE	0
GBR	1

### 3.2 Methodology

Qualitative Comparative Analysis (QCA) was first proposed by Charles Ragin in 1987 and has since developed into one of the main research methods in the field of social science. QCA is grounded in the mathematical principles of Boolean algebra and set theory, identifying combinations of conditions that influence the outcome variable (52). Compared to qualitative research and quantitative research, the QCA method has the following three distinctive characteristics: First, the QCA method is more suitable for research involving a moderate number of case studies. As per prevailing QCA research practices, the number of cases studied typically lies between 10 and 40, with conditional variables ranging from 4 to 6 (52). This roughly aligns with the number of cases and conditional variables in this article. Despite the presence of seven conditional variables in the current analytical framework of this article, there is a selection process for conditional variables in the QCA method, which is also applicable to this article. Second, the QCA method is proficient in analyzing complex variable combinations and identifying causal mechanisms. Public policies during the first wave of the COVID-19 pandemic have numerous influencing factors that make it difficult to explain policy outcomes with simple individual variables. Furthermore, the QCA method’s research conclusions are directed toward results from specific combinations of conditions, providing distinctive benefits in recognizing and confirming causal mechanisms. Third, since the QCA method is grounded in set theory, the variable relationships identified by QCA are asymmetric. To summarize, this article utilizes the QCA method and the analysis software fsQCA4.1 (53). Presently, QCA has evolved methodologies like crisp-set, fuzzy-set, and multi-value set analysis. Given that most conditional variables in this article are qualitative, especially those related to political factors, this article adopts crisp-set analysis in accordance with the general principles of the QCA method.

What needs illustration is that: Firstly, the QCA method’s inherent asymmetry in identifying causal mechanisms often leads researchers to focus solely on one outcome variable direction, either “yes” or “no.” Secondly, this article utilizes the crisp-set (binary variable) approach. This means the case set for non-implementation of lockdowns encompasses internal variations, represented by “0,” “1,” and “2” as sub-case sets. This diversity complicates identifying the combinations of conditions and causal mechanisms leading to the non-implementation of lockdown policies. Thirdly, the theoretical considerations of this article primarily arise from investigating public crisis management challenges attributed to uncertainties. Consequently, examining the “implementation of lockdown measures,” a policy option characterized by considerable intensity and controversy, aligns closely with the central research question of this article. Overall, the empirical analysis within this article will predominantly focus on the case set related to the “implementation of lockdown measures.”

### 3.3 Operationalizing the conditions

**Whether a former Eastern Bloc country (EB)**. These countries, influenced by their political culture and systems during the Cold War, place more emphasis on collectivism and authority in their political culture (54) and are more acceptable to regulation in the economic and social spheres compared to other European countries. The political heritage of the former Eastern Bloc countries could potentially facilitate smoother navigation through decision-making processes, particularly when implementing lockdown measures. This historical background may also contribute to a greater public understanding and support for such policies. We code “yes, a former Eastern Bloc country” as “1,” and “no, not a former Eastern Bloc country” as “0.”

**Severity of the Pandemic (SP)**. This article examines the severity of the pandemic in European countries prior to April 15, 2020. Typically, two fundamental indicators are essential in assessing the severity of the pandemic in a country. The first is the confirmed cases rate in the country, i.e., the proportion of confirmed cases to the total population. This indicator measures the spread of the virus in the country. The second is the COVID-19 death rates, which is the proportion of deaths from COVID-19 relative to the total number of confirmed cases. This indicator measures the severity of the threat to the lives and health of people in the country. The data on the number of confirmed cases and deaths are dependent on the cumulative figures starting from April 15, 2020, sourced from the official website of the WHO (3). The total population data is rooted in the 2020 population figures of each country, sourced from the official United Nations website (55). Since both of these indicators are crucial in measuring the severity of the pandemic, this article conducts a dichotomous K-Means Cluster Analysis centered around the diagnosis and death rates of European countries as of April 15, to provide a more comprehensive evaluation of the pandemic’s severity in each country. The outcomes of the cluster analysis are presented in Tables 3, 4. Stemmed from the cluster analysis, Cluster 1 has both higher diagnosis and death rates compared to Cluster 2, implying a greater severity of the pandemic in Cluster 1. Consequently, Cluster 1 is coded as “1,” and Cluster 2 as “0.”

**Table 3:** Summary of basic information on cluster analysis (SP).

Cluster	Counts	Percentage
cluster_1	8	30.77%
cluster_2	18	69.23%
total	26	100%

**Table 4:** Cluster analysis ANOVA difference comparison results (SP).

	Mean ± Standard deviation		F	p
	cluster_1 (*n* = 8)	cluster_2 (*n* = 18)		
Confirmed cases rates	0.00 ± 0.00	0.00 ± 0.00	28.037	0.000**
Deaths rates	0.12 ± 0.03	0.03 ± 0.02	74.618	0.000**

*p < 0.05; **p < 0.01.

**Economic situations (ES)**. Economic growth rate is an indicator of a country’s overall economic situation, and the unemployment rate serves as a crucial measure on the socio-political level to evaluate the economic and social pressures the government confronting with. Hence, this article will employ the 2019 economic growth rates (GDP growth rates) and unemployment rates of European countries to gage their economic situations. The economic growth rate data is sourced from the World Bank official website (56, 57),^1^ and the unemployment rate data from the OECD official website (58).^2^ This article undertakes a three-pointed cluster analysis of economic growth rates and unemployment rates. The analysis findings are shown in Tables 5, 6. From the analysis, it is evident that Cluster 1 has a higher unemployment rate and a lower economic growth rate compared to Cluster 2, while Cluster 3 has the highest unemployment rate. In consequence, the economic conditions of Cluster 1 and 3 are worse compared to Cluster 2. Consequently, Cluster 1 is coded as “1,” Cluster 2 as “0,” and Cluster 3 as “1.”

**Table 5:** Summary of basic information on cluster analysis (ES).

Cluster	Counts	Percentage
cluster_1	13	50.00%
cluster_2	11	42.31%
cluster_3	2	7.69%
Total	26	100%

**Table 6:** Cluster analysis ANOVA difference comparison results (ES).

	Mean ± Standard deviation			F	p
	cluster_1 (*n* = 13)	cluster_2 (*n* = 11)	cluster_3 (*n* = 2)		
Unemployment rate	5.94 ± 1.88	4.78 ± 1.54	16.00 ± 2.69	33.699	0.000**
GDP growth	1.82 ± 0.65	4.20 ± 0.71	1.94 ± 0.20	39.766	0.000**

*p < 0.05; **p < 0.01.

**State Fragility (SF)**. State Fragility denotes the presence of actual challenges and potential risks in a country regarding its state capabilities and legitimacy. The Center for Systemic Peace (CSP) published the State Fragility Index and Matrix 2018,^3^ collecting and coding data on the fragility risks to state capacity. According to the “State Fragility Index and Matrix 2018” data, this article assigns a code of “1” to countries with related risks and “0” to those without such risks.

**Party System (PS)**. The effective parties serve as an important indicator for assessing the real political influence exerted by parties within national parliaments.

The most common way to measure it is (59):


$$N_{L-T}=1/\overset{x}{\underset{1}{\sum }}S_{i}^{2}$$

When the number of effective parties is small, it indicates a greater concentration of party power in the national parliament; conversely, a larger number suggests more dispersion. The original data on the party system, forms of cabinet, and the ruling party’s ideology are all sourced from the ParlGov database (60). Generally, a value of $N_{L-T}\ll 2.5$ defines a two-party system, $2.5<N_{L-T}\ll 4.5$ as a moderate multiparty system, $N_{L-T}>4.5$ a polarized multiparty system (61). Thus, we code countries as “1” with $N_{L-T}\ll 4.5$ who have relatively centralized party power, while those with $N_{L-T}>4.5$ , indicating relatively dispersed party power, are coded as “0.”

**Forms of Cabinet (FC)**. The form of the cabinet primarily examines whether the ruling party is a majority party or part of a ruling coalition in the parliament. Thus, this article codes countries with the ruling party holding 0.5 or more of the parliamentary seats as “1,” and those with less than 0.5 as “0.”

**Ruling Party’s Ideology (PI)**. In this article, countries with a left-wing ruling party are coded as “1,” and those with a right-wing ruling party are coded as “0.” If the ruling party is a ruling coalition, the coding is weighted by the ideology and seats proportion of each party in the ruling coalition. In a nutshell, the coding of the relevant conditional variables and result variables in this article is as shown in Table 7.

**Table 7:** Conditions coding.

Country	EB	SP	ES	SF	PS	FC	PI	Lockdown
AUT	0	0	1	0	1	1	0	1
BEL	0	1	1	1	0	0	0	1
BGR	1	0	0	1	1	1	0	0
HRV	1	0	0	1	1	0	0	0
CYP	0	0	0	1	0	0	0	1
CZE	1	0	0	1	0	0	0	1
DNK	0	0	1	0	0	0	1	0
EST	1	0	0	0	1	1	0	0
FIN	0	0	1	0	0	1	1	0
FRA	0	1	1	1	1	1	0	1
DEU	0	0	1	1	0	1	0	0
GRC	0	0	1	1	1	1	0	1
HUN	1	0	0	0	1	1	0	0
IRL	0	1	0	0	0	0	0	1
ITA	0	1	1	1	1	1	1	1
LVA	1	0	1	0	0	1	0	0
LTU	1	0	0	1	0	1	1	0
NLD	0	1	1	0	0	1	0	0
POL	1	0	0	0	1	1	0	1
PRT	0	0	1	0	1	0	1	0
ROU	1	0	0	1	1	0	0	1
SVK	1	0	1	1	1	1	0	1
SVN	1	0	0	0	0	1	0	1
ESP	0	1	1	1	0	0	1	1
SWE	0	1	1	0	0	0	1	0
GBR	0	1	1	1	1	1	0	1

## 4 Results of the empirical analysis

Utilizing the QCA method, the article initially examines the necessary conditions for a country’s implementation of lockdown measures, setting the outcome variable to 1. The findings of the necessary condition analysis can be found in Table 8. As demonstrated in Table 8, no condition forms a necessary requirement for the outcome variable. Thus, this article incorporates all relevant conditional variables into the ensuing sufficient condition analysis.

**Table 8:** Analysis on the necessary conditions for the outcome “lockdown.”

	Consistency	Coverage
EB	0.36	0.45
SP	0.43	0.75
ES	0.57	0.53
SF	0.71	0.71
PF	0.57	0.62
FC	0.57	0.50
PI	0.14	0.29

Given that this article involves 26 cases, having four to five conditional variables is generally advisable. We analyze combinations containing both four and five conditional variables. Adhering to the principle of minimalist design and taking into account both consistency and coverage, the following four conditional variables are deemed the most appropriate for the analysis. The truth table is presented in Table 9. The threshold for case inclusion in this article is set at 1. Following QCA practices, this article establishes a consistency threshold of 0.8 for the set “lockdown.” Combinations of conditions with a value greater than 0.8 are coded as “1,” and all others as “0.” In the course of analysis, this article ultimately incorporates four conditional variables, logically leading to 16 distinct condition combinations. From the 26 cases in this article, the truth table created encompasses 14 out of the 16 possible condition combinations, leaving only two logical remainders.

**Table 9:** Truth table for the outcome “lockdown.”

ES	SF	PS	FC	N	Lockdown	Case	Consistency
1	1	1	1	5	1	FRA, GRC, ITA, SVK, GBR	1
0	1	0	0	2	1	CYP, CZE	1
1	1	0	0	2	1	BEL, ESP	1
0	0	0	0	1	1	IRL	1
0	0	0	1	1	1	SVN	1
1	0	1	1	1	1	AUT	1
0	1	1	0	2	0	HRV, ROU	0.5
0	0	1	1	3	0	EST, HUN, POL	0.3
1	0	0	1	3	0	FIN, LVA, NLD	0
1	0	0	0	2	0	DNK, SWE	0
1	0	1	0	1	0	PRT	0
0	1	0	1	1	0	LTU	0
1	1	0	1	1	0	DEU	0
0	1	1	1	1	0	BGR	0

Owing to the asymmetric causal relationship delineated by the QCA method, and constrained by the scope of the research focus and the article’s length, the subsequent analysis will solely concentrate on the cases of implementing lockdown measures. Drawn from the truth table, this article uses fsQCA4.1 software to solve for the conditions combinations of a country implementing lockdown measures. Given the limited number of cases and the minimal logical remainders in the truth table, the parsimonious, intermediate, and complex solutions produced in this analysis are aligned. The solution can be found in Table 10.

**Table 10:** Solution of sufficient conditions for the outcome “lockdown.”

Sufficient combination	Consistency	Coverage	Case
~ ES* ~ SF* ~ PS	1	0.142857	IRL, SVN
SF* ~ PS* ~ FC	1	0.285714	BEL, CYP, CZE, ESP
ES *PS* FC	1	0.428571	AUT, FRA, GRC, ITA, SVK, GBR

Solution consistency: 0.857143; solution coverage: 1.

According to the solution results, there are three types of conditions combinations that lead a country to implement lockdown measures, signifying three fundamental patterns among countries that enforce these measures.

Model One: ~ES * ~ SF * ~ PS. This model corresponds to the cases of Ireland and Slovenia. This means that under conditions of a strong economic situation, absence of the state fragility risk, and a decentralized party system, countries will implement lockdown measures. This demonstrates that lockdown measures, being impactful and highly urgent public measures, are implemented in contexts where overall political, economic, and social situations are favorable. In such scenarios, the economic and social costs of lockdown measures are relatively minor, and there is less policy divergence among major parties. Even in a setting of decentralized party power, timely adoption of lockdown measures to combat the pandemic’s spread is still feasible. The Government of Ireland is a majority coalition government of Fianna Fáil, Fine Gael and Green Party. All three parties supported implementing lockdown policies at the early stage of the pandemic. As early as March 12, 2020, the Irish government closed schools, childcare facilities, and cultural institutions, subsequently upgrading lockdown measures several times. The government’s stance on lockdown policies remained unchanged even with the inauguration of a new Prime Minister from Fianna Fáil, a member of the governing coalition. During this period, Sinn Féin, the largest opposition party, also supported lockdown measures. Sinn Féin leader Mary Lou McDonald criticized the neighboring British government’s early abandonment of lockdown measures, calling them “dangerous and reckless (62).

Model Two: SF * ~ PS * ~ FC. This model corresponds to the cases of Belgium, Cyprus, the Czech Republic, and Spain. Under conditions of state fragility risk, countries will still implement timely lockdown measures, even if they have a dispersed party system and a minority party rule. These countries will still implement lockdown measures despite potentially facing more procedural interference in the public policy process and greater political resistance in parliamentary politics. This implies that these countries are sensitive to their own state fragility risks and consider the prevention of the pandemic’s spread and the exacerbation of internal social risks as key factors in actively pursuing lockdown measures. For example, despite facing considerable political pressure, Spain began implementing lockdown measures on March 14, 2020. Although these measures were later ruled unconstitutional by the Spanish Supreme Court in 2021, they proved effective in curbing the spread of the pandemic in its early stages (63). However, due to Spain’s significant regionalism, the nationwide lockdown policy was executed differently across various regions, which leads to notable differences in the effectiveness of pandemic control (64, 65).

Model Three: ES * PS * FC. This model corresponds to the cases of Austria, France, Greece, Italy, Slovakia, and the UK, which are positive examples in this article. In scenarios where the economic situation is poor but the party system is more centralized and the cabinet is led by a majority party or coalition, these countries have enacted lockdown measures. A centralized party system and cabinet authority lead to smoother development and execution of public policies. Meanwhile, the poor economic situation heightens these countries’ sensitivity to the severe threats that the further spread of the pandemic may pose to their national economies. Although implementing lockdown policies at the early stage of the pandemic entailed high economic and social costs in the short term, the recent literature have found that the relationship between lockdown policies and economic costs is not linear. If early lockdown measures effectively control the spread of the pandemic, these economic costs can be quickly offset (66). Moreover, a scientifically designed combination of lockdown policies can effectively reduce the economic costs associated with such measures (67). Under these circumstances, quickly controlling the pandemic and stabilizing the socio-economic situation becomes the primary choice of the major parties, especially the ruling parties in these countries. During the early stages of the pandemic, Italy was governed by a grand coalition primarily comprising the Five Star Movement and the Democratic Party, which held a majority of seats in both the Chamber of Deputies and the Senate. To effectively control the outbreak, the Italian government implemented some of the earliest and strictest lockdown policies in Europe, with the main ruling parties actively supporting and cooperating with these measures. On February 21, 2020, Italy imposed “red zone” lockdowns on parts of Lombardy and Venice. This lockdown policy was extended nationwide on March 9, making Italy the first European country to implement a nationwide lockdown (68). Although the lockdown imposed significant economic pressure on Italy (69), the government deemed it a necessary measure to protect the health of all citizens (70).

Among the cases, 14 countries enacted lockdown measures. The three models described above accounted for 12, with Romania and Poland remaining unexplained. Observing the fundamental circumstances of both countries, they exhibit several similarities. On one hand, both countries experienced lower severity of the pandemic and better economic situations, indicating lesser socio-economic and pandemic pressures for them. Conversely, both countries are ruled by majority parties with fewer effective parties, signifying that their political power structures are relatively centralized. Under such conditions, both have implemented lockdown measures, which are closely related to the policy propositions of the ruling party, and the successful implementation of the lockdown measures is mainly due to its relatively centralized political power structure.

## 5 Conclusion and discussion

From the essential insights of the QCA empirical analysis, this article primarily derives the following three key conclusions:

Firstly, countries with comparatively centralized political power are more inclined to enforce lockdown measures. The criterion for coding the party system as “1” is having less than 4.5 effective parties, aligning with a two-party or moderate multiparty system. The guideline for coding forms of cabinet as “1” is governance by either a majority party or a majority coalition. The combination of “PS * FC” suggests that in these countries, both at the level of the parliament, which holds legislative authority, and the cabinet, which holds executive authority, only a limited number of parties wield real power, indicating more centralized political power. At the first wave of the COVID-19 pandemic, there were no effective therapeutics in the management of the pandemic. Hence, the COVID-19 pandemic represented a severe public health crisis for countries at that time, making the implementation of mobility restriction policies the most straightforward and effective strategy (71). Yet, the implementation of lockdown measures might entail considerable socio-economic pressures and ideological disputes. Centralized political power implies that the ruling party is more capable of implementing lockdown measures while also bearing greater political responsibility. The ruling party is also more politically motivated to quickly control the pandemic. For minority governments in multiparty countries, promptly advancing the policy agenda presents a challenge (35). Based on the cases in this article, “PS*FC” can provide elaboration for 57% of cases. From the categorical summary in Table 11, it demonstrates that among the 10 countries with relatively centralized political power, seven implemented lockdown measures, a rate of 70%. For the 16 countries with relatively dispersed political power, only seven implemented lockdown measures, a rate of 44%. The possibility of countries with relatively centralized political power implementing lockdown measures is higher than in other countries.

**Table 11:** Categorical summary of “PS* FC” and “lockdown.”

Conditions	Counts	N	Percentage
PS * FC	10	7	70%
~ PS + ~ FC	16	7	44%

Secondly, countries facing state fragility risks are more inclined to enforce lockdown measures in contexts where political power is relatively dispersed. Upon examining countries with relatively centralized political power, it becomes evident that for those with dispersed political power, their inherent state fragility risks significantly influence the timely implementation of lockdown measures. As indicated in Table 12, among the 16 countries with more dispersed political power, eight each either face or do not face state fragility risks. In the group with state fragility risks, five countries implemented lockdown measures, accounting for 63%; in contrast, only two of the countries without such risks did so, amounting to 25%. The probability of enacting lockdown measures is notably higher in the former compared to the latter. Even with relatively dispersed political power, all political entities engaged in the public policy process must jointly address the political risks and state capacity risks faced by the country. The ruling party, bearing the primary political responsibility, is also more motivated to implement lockdown measures to prevent the exacerbation of political risks that could result from the further spread of the virus. Especially as a key component of lockdown policies, road traffic control can significantly increase the costs of political mobilization, thereby reducing the risk of social unrest. When combined with economic support measures, lockdown policies can further reduce political risk (72). In this regard, lockdown measures are not only a means to control the pandemic but also a strategy to prevent social risks associated with the pandemic from transforming into political risks.

**Table 12:** Categorical summary of “(~ PS + ~ FC)*SF” and “lockdown.”

Conditions	Counts	N	Percentage
(~ PS + ~ FC)* SF	8	5	63%
(~ PS + ~ FC)* ~ SF	8	2	25%

In the process of elucidating the two research conclusions mentioned above, this article examines the variance in “implementation of lockdown measures” across 26 case countries, each characterized by distinct political power structures. The core contributions of this article are the identification of three critical factors—"party system,” “forms of cabinet,” and “state fragility risk”—and their impacts on the implementation of lockdown measures. In the aforementioned comparative analysis, this article has effectively incorporated instances of countries that abstained from implementing lockdown measures, thereby facilitating a control group analysis based on the three primary conditions. Additionally, the comparative analysis predicated on these three conditions serves as a robustness check for the QCA employed in this article.

Thirdly, factors like political heritage, the severity of the pandemic, and the ruling party’s ideology play a minor role in determining the implementation of lockdown measures.

Regarding the political heritage, where other conditions are similar, the distinction in lockdown measures choices between former Eastern Bloc countries and non-Eastern Bloc countries is minimal, suggesting that political heritage is not an important determinant in decision-making. Alongside the similarly minor role of the ruling party’s ideology, it can be inferred that the cultural divergence in European countries concerning pandemic management is relatively subdued compared to practical interest considerations (73).

Concerning the severity of the pandemic, it represents the degree of the public health crisis it causes, yet the severity itself has not been a significant factor in countries implementing lockdown measures. This suggests that decision-makers in European countries are more strongly influenced by political factors than by the pandemic itself or the expert opinions of professional health organizations (74).

Considering the ruling party’s ideology, several studies have noted that political party ideology in the United States has impacted state-level pandemic control policies (33, 75) but this influence is not significant at the national level in European countries. This is partly due to the fact that European countries primarily formulated policies at the national level during the first wave; moreover, the widespread multi-party systems in Europe have lessened the influence of ideological divisions in decision-making during the public health crisis. Additionally, the involvement of the EU in pandemic policy has increased the consistency of pandemic policies across countries (76) and reduced the influence of individual national parties.

To summarize the three main conclusions above, the formulation and implementation of policies for preventing and controlling the COVID-19 pandemic should have been a public health issue and a matter of public policy, as a policy “trial” for responding to a severe public health crisis. However, as discussed at the beginning of this article, the balance between economic and social aspects, pandemic prevention, and the choice between development and health may become the main considerations of countries in pandemic prevention. In fact, whether countries implemented lockdown measures during the first wave was more of a political issue. On the one hand, it was the influence of the political power structure of each country on the feasibility of implementing the corresponding lockdown measures, and only countries with relatively centralized political power were more likely to implement lockdown measures. On the other hand, it was the alertness of countries to the political risk that the pandemic may bring that made the political parties more inclined to adopt lockdown measures to control the spread of the pandemic as soon as possible, even if the political power was relatively decentralized. Reviewing the political vacillations of European countries in mobility restriction policies during this period is also of significance to our understanding of the formation of differences in the spectrum of mobility restriction policies across the globe.

We must point out that the Policy-Making Process and Evaluating Policy Performance are different aspects of Policy Analysis (77), and this article primarily focuses on the Policy-Making Process. In the early stages of the COVID-19 pandemic, due to the lack of targeted measures and treatments, traditional non-pharmaceutical interventions for infectious diseases were not only the only feasible option for controlling the spread of the virus but were also proven effective by many studies during this phase. However, implementing lockdown policies required a difficult balance between power and freedom, and health and development, creating a complex decision-making environment for policymakers. This provides a realistic foundation for studying the decision-making process and influencing factors of lockdown policies. We also acknowledge that it is meaningful to continue deepening our understanding of the effectiveness of lockdown policies at different stages of the COVID-19 pandemic, including the early stage. Moreover, the series of political factors we focus on not only affect the decision-making process of COVID-19 policies but also their outcomes, which will be the direction for our future research.

## Data Availability

The original contributions presented in the study are included in the article/Supplementary material, further inquiries can be directed to the corresponding author.

## Footnotes

^1^Data Source: World Bank national accounts data, and OECD National Accounts data files.

^2^Data Source: Organization for Economic Co-operation and Development (OECD).

^3^Technical Notes to the State Fragility Index and Matrix 2018: The State Fragility Index and Matrix 2018 lists all independent countries in the world in which the total country population was greater than 500,000 in 2018 (167 countries). At the end of the year in 2018, the Fragility Matrix scored each country on both effectiveness and legitimacy in four performance dimensions: security, political, economic, and social. Each of the Matrix’s indicators is rated on a four-point fragility scale: 0 “no fragility “1 “low fragility “2 “medium fragility,” and 3 “high fragility” with the exception of the Economic Effectiveness indicator, which is rated on a five-point fragility scale (including 4 “extreme fragility”). The State Fragility Index, then, combined the scores on the eight indicators and ranged them from 0 “no fragility” to 25 “extreme fragility.” A country’s fragility is closely associated with its state’s ability to: manage conflict make and implement public policy: deliver essential services. A country’s fragility is also measured by its systemic resilience in maintaining system coherence, cohesion, and quality of life; it is ability to respond effectively to challenges and crises; and whether or not it has sustainable, progressive development.

## References

[1] Beck U. Risk society: Towards a new modernity. Thousand Oaks, CA: Sage (1992).

[2] Whipple C. Dealing with uncertainty about risk in risk management In: Lave LB, editor. Risk assessment and management. Boston, MA: Springer US (1987). 529–36.

[3] World Health Organization. WHO COVID-19 dashboard. (2023). Available at: https://data.who.int/dashboards/covid19/cases?n=c (Accessed July 23, 2024).

[4] World Health Organization. Statement on the second meeting of the international health regulations (2005) emergency committee regarding the outbreak of novel coronavirus (2019-Ncov). (2020). Available at: https://www.who.int/news/item/30-01-2020-statement-on-the-second-meeting-of-the-international-health-regulations-(2005)-emergency-committee-regarding-the-outbreak-of-novel-coronavirus-(2019-ncov) (Accessed July 23, 2024).

[5] World Health Organization. Who director-General's opening remarks at the media briefing on Covid-19-11 march 2020. (2020). Available at: https://www.who.int/director-general/speeches/detail/who-director-general-s-opening-remarks-at-the-media-briefing-on-covid-19---11-march-2020 (Accessed July 23, 2024).

[6] France 24. UN chief says coronavirus worst global crisis since world war II. (2020). Available at: https://www.france24.com/en/20200401-un-chief-says-coronavirus-worst-global-crisis-since-world-war-ii (Accessed July 23, 2024).

[7] Ayouni I Maatoug J Dhouib W Zammit N Fredj SB Ghammam R. Effective public health measures to mitigate the spread of Covid-19: a systematic review. BMC Public Health. (2021) 21:1–14. doi: 10.1186/s12889-021-11111-1, PMID: 34051769 PMC8164261

[8] Palladino R Bollon J Ragazzoni L Barone-Adesi F. Effect of implementation of the lockdown on the number of Covid-19 deaths in four European countries. Disaster Med Public Health Prep. (2021) 15:e40–2. doi: 10.1017/dmp.2020.433, PMID: 33143797 PMC7943955

[9] Chung HW Apio C Goo T Heo G Han K Kim T. Effects of government policies on the spread of Covid-19 worldwide. Sci Rep. (2021) 11:20495. doi: 10.1038/s41598-021-99368-9, PMID: 34650119 PMC8516948

[10] Pagliari C. The ethics and value of contact tracing apps: international insights and implications for Scotland’s Covid-19 response. J Glob Health. (2020) 10:020103. doi: 10.7189/jogh.10.020103, PMID: 33110502 PMC7510337

[11] Gopinath G. The great lockdown: Worst economic downturn since the great depression. (2020). Available at: https://www.imf.org/en/Blogs/Articles/2020/04/14/blog-weo-the-great-lockdown-worst-economic-downturn-since-the-great-depression (Accessed July 23, 2024).

[12] OECD. Our global reach. (2023). Available at: https://www.oecd.org/about/members-and-partners/ (Accessed July 23, 2024).

[13] World Health Organization. Statement on the second meeting of the international health regulations (2005) emergency committee regarding the outbreak of novel coronavirus (2019-nCoV). (2023). Available at: https://www.who.int/news/item/30-01-2020-statement-on-the-second-meeting-of-the-international-health-regulations-(2005)-emergency-committee-regarding-the-outbreak-of-novel-coronavirus-(2019-ncov) (Accessed July 23, 2024).

[14] Araral E Fritzen S Howlett M Ramesh M Wu X. Routledge handbook of public policy. New York: Routledge (2012).

[15] Cooper J Dimitriou N Arandjelovíc O. How good is the science that informs government policy? A lesson from the UK’s response to 2020 Cov-2 outbreak. J Bioethic Inq. (2021) 18:561–8. doi: 10.1007/s11673-021-10130-2 PMC8515150 34648101

[16] Rubin O Errett NA Upshur R Baekkeskov E. The challenges facing evidence-based decision making in the initial response to Covid-19. Scand J Public Health. (2021) 49:790–6. doi: 10.1177/1403494821997227, PMID: 33685289

[17] Dattner I Gal R Goldberg Y Goldshtein I Huppert A Kenett RS. The role of statisticians in the response to Covid-19 in Israel: a holistic point of view. Isr J Health Policy Res. (2022) 11:1–9. doi: 10.1186/s13584-022-00531-y 35443682 PMC9019798

[18] Bavel JJV Baicker K Boggio PS Capraro V Cichocka A Cikara M. Using social and Behavioural science to support Covid-19 pandemic response. Nat Hum Behav. (2020) 4:460–71. doi: 10.1038/s41562-020-0884-z, PMID: 32355299

[19] Rhodes T Lancaster K. Mathematical models as public troubles in Covid-19 infection control: following the numbers. Health Sociol Rev. (2020) 29:177–94. doi: 10.1080/14461242.2020.1764376, PMID: 33411652

[20] Bershteyn A Kim H-Y Braithwaite RS. Real-time infectious disease modeling to inform emergency public health decision making. Annu Rev Public Health. (2022) 43:397–418. doi: 10.1146/annurev-publhealth-052220-093319, PMID: 34995131

[21] Green L Ashton K Azam S Dyakova M Clemens T Bellis MA. Using health impact assessment (HIA) to understand the wider health and well-being implications of policy decisions: the Covid-19 ‘staying at home and social distancing Policy’in Wales. BMC Public Health. (2021) 21:1456. doi: 10.1186/s12889-021-11480-7, PMID: 34315469 PMC8313659

[22] Means AR Wagner AD Kern E Newman LP Weiner BJ. Implementation science to respond to the Covid-19 pandemic. Front Public Health. (2020) 8:462. doi: 10.3389/fpubh.2020.00462, PMID: 32984248 PMC7493639

[23] Pearse H. Deliberation, citizen science and Covid-19. Polit Q. (2020) 91:571–7. doi: 10.1111/1467-923X.12869, PMID: 32836410 PMC7361354

[24] Rode DC Fischbeck PS. On ambiguity reduction and the role of decision analysis during the pandemic. Risk Anal. (2021) 41:721–30. doi: 10.1111/risa.13705, PMID: 33534949 PMC8013914

[25] Sözen ME Sarıyer G Ataman MG. Big data analytics and Covid-19: investigating the relationship between government policies and cases in Poland, Turkey and South Korea. Health Policy Plan. (2022) 37:100–11. doi: 10.1093/heapol/czab096, PMID: 34365501 PMC8385927

[26] Bel G Gasulla Ó Mazaira-Font FA. The effect of health and economic costs on Governments' policy responses to Covid-19 crisis under incomplete information. Public Adm Rev. (2021) 81:1131–46. doi: 10.1111/puar.13394, PMID: 34226767 PMC8242661

[27] Kelly P Hofbauer S Gross B. Renegotiating the public good: responding to the first wave of Covid-19 in England, Germany and Italy. Eur Educ Res J. (2021) 20:584–609. doi: 10.1177/14749041211030065

[28] Reich MR. Pandemic governance in Japan and the United States: the control-tower metaphor. Health Syst Reform. (2020) 6:e1829314. doi: 10.1080/23288604.2020.1829314, PMID: 33236940

[29] Comfort LK Kapucu N Ko K Menoni S Siciliano M. Crisis decision-making on a global scale: transition from cognition to collective action under threat of Covid-19. Public Adm Rev. (2020) 80:616–22. doi: 10.1111/puar.13252, PMID: 32836462 PMC7300963

[30] Davies SE Wenham C. Why the Covid-19 response needs international relations. Int Aff. (2020) 96:1227–51. doi: 10.1093/ia/iiaa135, PMID: 34191916 PMC7797718

[31] Tesche T. Pandemic politics: the European Union in times of the coronavirus emergency. J Common Mark S. (2022) 60:480–96. doi: 10.1111/jcms.13303

[32] Hafsi T Baba S. Exploring the process of policy overreaction: the Covid-19 lockdown decisions. J Manag Inq. (2023) 32:152–73. doi: 10.1177/10564926221082494, PMID: 36814993 PMC9936179

[33] Shvetsova O Zhirnov A Giannelli FR Catalano MA Catalano O. Governor's party, policies, and Covid-19 outcomes: further evidence of an effect. Am J Prev Med. (2022) 62:433–7. doi: 10.1016/j.amepre.2021.09.003, PMID: 34756754 PMC8502787

[34] Migone AR. Trust, but customize: Federalism’s impact on the Canadian Covid-19 response. Polic Soc. (2020) 39:382–402. doi: 10.1080/14494035.2020.1783788, PMID: 35039727 PMC8754695

[35] Luyten J Schokkaert E. Belgium's response to the Covid-19 pandemic. Health Econ Policy L. (2022) 17:37–47. doi: 10.1017/S1744133121000232, PMID: 34219632 PMC8280466

[36] Moon W-K Atkinson L Kahlor LA Yun C Son HUS. Political partisanship and Covid-19: risk information seeking and prevention behaviors. Health Commun. (2022) 37:1671–81. doi: 10.1080/10410236.2021.1912948 33906522

[37] Cronert A. Precaution and proportionality in pandemic politics: democracy, state capacity, and Covid-19-related school closures around the world. J Public Policy. (2022) 42:705–29. doi: 10.1017/S0143814X22000101

[38] Cai C Jiang W Tang N. Campaign-style crisis regime: how China responded to the shock of Covid-19. Policy Stud. (2022) 43:599–619. doi: 10.1080/01442872.2021.1883576

[39] Gao J Zhang P. China's public health policies in response to Covid-19: from an “authoritarian” perspective. Front Public Health. (2021) 9:756677. doi: 10.3389/fpubh.2021.756677 34976920 PMC8714736

[40] Mao Y. Political institutions, state capacity, and crisis management: a comparison of China and South Korea. Int Polit Sci Rev. (2021) 42:316–32. doi: 10.1177/0192512121994026

[41] Zhang J Zhang R. Covid-19 in China: power, transparency and governance in public health crisis. Health. (2020) 8:288. doi: 10.3390/healthcare8030288, PMID: 32842607 PMC7551406

[42] Zysset A Vidal-Marti N. ‘Constitutionalism and Covid-19: broadening the Lens’ with jus Cogens. Jus Cogens. (2022) 4:203–5. doi: 10.1007/s42439-022-00067-4

[43] Berger P. Proportionality, evidence and the Covid-19-jurisprudence in Germany. Eur J Secur Res. (2022) 7:211–36. doi: 10.1007/s41125-022-00087-7

[44] Hervey TK Röttger-Wirtz S. The European Union: legal response to Covid-19 In: King J Ferraz OL, editors. The Oxford compendium of National Legal Responses to Covid-19. Oxford: Oxford University Press (2022)

[45] Grogan J Donald A. Routledge handbook of law and the Covid-19 pandemic. New York: Routledge (2022).

[46] Béland D Dinan S Rocco P Waddan A. Covid-19, poverty reduction, and partisanship in Canada and the United States. Polic Soc. (2022) 41:291–305. doi: 10.1093/polsoc/puac002

[47] Easton D. A systems analysis of political life. New York: John Wiley & Sons, Inc (1965).

[48] GBD 2016. Healthcare access and quality collaborators. Measuring performance on the healthcare access and quality index for 195 countries and territories and selected subnational locations: a systematic analysis from the global burden of disease study 2016. Lancet. (2018) 391:2236–71. doi: 10.1016/s0140-6736(18)30994-2 29893224 PMC5986687

[49] United Nations. World population prospects: 2019 revision. (2019). Available at: https://population.un.org/wpp2019/ (Accessed July 23, 2024).

[50] CSP. Polity5 Dataset Version 2018 <P5v2018 and P5v2018d>. (2018). Available at: https://www.systemicpeace.org/polityproject.html (Accessed July 23, 2024).

[51] Piccoli L Ader L Hoffmeyer-Zlotnik P Mittmasser C Pedersen O Pont A. Mobility and border control in response to the Covid-19 outbreak dataset. (2020). Available at: https://hdl.handle.net/1814/68358 (Accessed July 23, 2024).

[52] Rihoux B Ragin CC. Configurational comparative methods: Qualitative comparative analysis (Qca) and related techniques. Thousand Oaks, California: Sage Publications (2008).

[53] Ragin CC. UC Irvine School of social science. Fs/Qca Software (2023). Available at: https://sites.socsci.uci.edu/~cragin/fsQCA/software.shtml (Accessed July 23, 2024).

[54] Huskey E. Authoritarian leadership in the post-communist world. Daedalus. (2016) 145:69–82. doi: 10.1162/DAED_a_00398

[55] United Nations. World population prospects: 2019 revision. (2019). Available at: https://population.un.org/wpp2019/ (Accessed July 23, 2024).

[56] The World Bank. World Bank National Accounts Data. (2023). Available at: https://data.worldbank.org/ (Accessed July 23, 2024).

[57] OECD. National Accounts. (2023). Available at: https://www.oecd.org/sdd/na/ (Accessed July 23, 2024).

[58] OECD. Organisation for economic co-operation and development (Oecd). (2023). Available at: https://www.oecd.org/ (Accessed July 23, 2024).

[59] Laakso M Taagepera R. “Effective” number of parties: a measure with application to West Europe. Comp Polit Stud. (1979) 12:3–27. doi: 10.1177/001041407901200101

[60] Döring H Manow P. Parliament and Government Composition Database (Parlgov): Information on Parties, Elections and Cabinets in Modern Democracies. (2012). Available at: https://www.researchgate.net/publication/228918733_Parliament_and_government_composition_database_ParlGov (Accessed July 23, 2024).

[61] Sartori G. Parties and party systems: A framework for analysis. Colchester: ECPR Press (2005).

[62] ITV Consumer. Sinn Féin leader brands UK Covid-19 response ‘dangerous’. (2020). Available at: https://www.itv.com/news/utv/2020-03-16/sinn-fein-leader-brands-uk-covid-19-response-dangerous (Accessed July 23, 2024).

[63] News Desk. Spain’s top court rules that state of alarm lockdown was ‘unconstitutional’. (2021). Available at: https://www.spainenglish.com/2021/07/15/spain-top-court-rules-state-of-alarm-lockdown-unconstitutional/ (Accessed July 23, 2024).

[64] Galindo Caldés R Tort Donada J Santasusagna RA. Administrative boundaries and Covid-19: the case of Catalonia, Spain In: Nunes Silva C, editor. Local government and the Covid-19 pandemic: A global perspective. Cham: Springer International Publishing (2022). 247–75.

[65] Siqueira C Freitas YNL Cancela MC Carvalho M Oliveras-Fabregas A de Souza DLB. The effect of lockdown on the outcomes of Covid-19 in Spain: an ecological study. PLoS One. (2020) 15:e0236779. doi: 10.1371/journal.pone.0236779, PMID: 32726363 PMC7390404

[66] Arias JE Fernández-Villaverde J Rubio-Ramírez JF Shin M. The causal effects of lockdown policies on health and macroeconomic outcomes. Am Econ J-Macroecon. (2023) 15:287–319. doi: 10.1257/mac.20210367

[67] Goldsztejn U Schwartzman D Nehorai A. Public policy and economic dynamics of Covid-19 spread: a mathematical modeling study. PLoS One. (2020) 15:e0244174. doi: 10.1371/journal.pone.0244174, PMID: 33351835 PMC7755180

[68] Nytimes. World Europe Italy Lockdown Coronavirus. (2020). Available at: https://www.nytimes.com/2020/03/09/world/europe/italy-lockdown-coronavirus.html (Accessed July 23, 2024).

[69] Amaro S. Italy vows to implement ‘a massive shock therapy’ against the coronavirus. (2020). Available at: https://www.cnbc.com/2020/03/09/italy-wants-more-public-spending-to-fight-coronavirus-amid-lockdown.html (Accessed July 23, 2024).

[70] BBC. World Europe. (2020). Available at: https://www.bbc.com/news/world-europe-51810673 (Accessed July 23, 2024).

[71] Cascini F Failla G Gobbi C Pallini E Hui J Luxi W. A cross-country comparison of Covid-19 containment measures and their effects on the epidemic curves. BMC Public Health. (2022) 22:1765. doi: 10.1186/s12889-022-14088-7, PMID: 36115936 PMC9482299

[72] Wood R Reinhardt GY Rezaeedaryakenari B Windsor LC. Resisting lockdown: the influence of Covid-19 restrictions on social unrest. Int Stud Q. (2022) 66:sqac015. doi: 10.1093/isq/sqac015

[73] Bojar A Kriesi H. Policymaking in the Eu under crisis conditions: Covid and refugee crises compared. Comp Eur Polit. (2023) 1-21:427–47. doi: 10.1057/s41295-023-00349-1

[74] McConnell A Stark A. Understanding policy responses to Covid-19: the stars Haven’t fallen from the sky for scholars of public policy. J Eur Public Policy. (2021) 28:1115–30. doi: 10.1080/13501763.2021.1942518

[75] Adolph C Amano K Bang-Jensen B Fullman N Magistro B Reinke G. The pandemic policy U-turn: partisanship, public health, and race in decisions to ease Covid-19 social distancing policies in the United States. Perspect Polit. (2022) 20:595–617. doi: 10.1017/S1537592721002036

[76] Quaglia L Verdun A. The Covid-19 pandemic and the European Union: politics, policies and institutions. J Eur Public Policy. (2023) 30:599–611. doi: 10.1080/13501763.2022.2141305

[77] Dunn WN. Public policy analysis: An integrated approach. New York: Routledge (2015).

